# Adaptation of CUT&RUN for use in African trypanosomes

**DOI:** 10.1371/journal.pone.0292784

**Published:** 2023-11-21

**Authors:** Geneva Miller, Lindsey M. Rollosson, Carrie Saada, Serenity J. Wade, Danae Schulz

**Affiliations:** Harvey Mudd College, Claremont, CA, United States of America; Columbia University Irving Medical Center, UNITED STATES

## Abstract

This Cleavage Under Targets and Release Using Nuclease (CUT&RUN) protocol produces genomic occupancy data for a protein of interest in the protozoan parasite *Trypanosoma brucei*. The data produced is analyzed in a similar way as that produced by ChIP-seq. While we describe the protocol for parasites carrying an epitope tag for the protein of interest, antibodies against the native protein could be used for the same purpose.

## Introduction

Mapping interactions between protein and DNA is critical for understanding many features of gene regulation. A large number of methods have been developed over the years to map these interactions, including but not limited to DNA footprinting, Chromatin ImmunoPrecipitation (ChIP), MNase-Seq and 3C (reviewed in [1]). The advent of high-throughput sequencing has allowed these tools to be combined with DNA sequencing to produce high resolution maps of protein binding sites and histone modifications throughout the genome. ChIP protocols can be performed with [2] or without [3] crosslinking, but crosslinking is sometimes necessary to assay transient or lower affinity interactions between DNA and the protein of interest. However, crosslinking can have drawbacks in some cases, including epitope masking and false positive binding sites [4–8].

To circumvent these issues, Peter Skene and Steven Henikoff developed Cleavage Under Targets and Release Using Nuclease (CUT&RUN), which does not include a crosslinking step and has the additional advantage of being faster than previous ChIP-based methods [9]. Originally used in yeast and mammalian cells, the method has since been widely adopted. In brief, cells are permeabilized and an antibody is added against the protein of interest. Following binding of this antibody to its target protein, a protein is added that includes a fusion of protein A to micrococcal nuclease. The fusion protein binds to the antibody bound to the target protein and micrococcal nuclease cleaves the DNA surrounding the binding site. Because the cleaved DNA is small, it can diffuse out through the nuclear pores. Thus, harvesting the supernatant following cleavage results in enrichment of DNA bound to the protein of interest, which can be sequenced to map binding sites genome-wide.

We recently adapted CUT&RUN for use in the protozoan parasite *Trypanosoma brucei*. *T*. *brucei* is the causative agent of Human African Trypanosomiasis (sleeping sickness) and Animal African Trypanosomiasis (nagana). It is transmitted to a mammalian host through the bite of a tsetse fly, and once in the bloodstream it lives extracellularly until it can be transmitted back to the tsetse via a bloodmeal. *T*. *brucei* is a particularly interesting model for gene regulation in a eukaryote because it diverged quite early from other well studied model organisms and has a number of unusual gene regulatory features. Genes in *T*. *brucei* are organized in polycistronic units, but genes within one unit are not necessarily functionally related (reviewed in [10–13]). While *T*. *brucei* harbors histones and histone modifications, both the histones and the modifications they contain diverge from other systems where these are known to be well conserved [14,15]. Finally, the *T*. *brucei* genome lacks obvious DNA sequence-specific transcription factors or binding sites, and post-transcriptional regulation drives many of the changes in transcript levels [11]. Because *T*. *brucei* is an early branching excavate, the study of its unusual gene regulatory features can lend insight into how gene regulation evolved across diverse biological organisms.

In a recent study, we used CUT&RUN to interrogate the occupancy of the chromatin interacting reader protein TbBdf3 [16], which binds to acetylated lysine residues and localizes to regions marked by H4K10ac [17]. Bromodomain proteins are important gene regulatory factors in many well-studied systems [18–20]. In *T*. *brucei*, Bdf3 localizes to transcription start sites and inhibition of Bdf3 by RNAi results in transcript changes that mirror those that occur as parasites transition from the bloodstream to the fly midgut [21]. We previously induced differentiation of bloodstream form parasites to the procyclic form that resides in the fly midgut and performed CUT&RUN with tagged Bdf3 protein to quantify its occupancy at binding sites throughout differentiation. We showed that occupancy of Bdf3 at most binding sites was dynamic during differentiation, and that a new site of Bdf3 occupancy formed near the procyclin gene locus [16]. A detailed analysis for bloodstream Bdf3 occupancy identified by ChIP-seq versus bloodstream Bdf3 occupancy identified by CUT&RUN is presented in Ashby et al [16]. 76% of divergent strand switch regions fell within 5kb of a Bdf3 occupied site in bloodstream parasites [16].

Key to adapting CUT&RUN for use in *T*. *brucei* was the development of a flow cytometry assay to assess permeabilization of the cell. To do this, we first permeabilized the parasite cells using a number of different protocols. We then incubated the permeabilized cells with a rabbit antibody against the histone protein H3. After washing the anti-H3 antibody away, we used an anti-rabbit IgG fluorescent secondary antibody and analyzed the cells by flow cytometry. This allowed us to quantify the number of successfully permeabilized cells within a population while also measuring whether the cells remained intact. In contrast to the original protocols that used digitonin in mammalian and yeast cells [9], we found that incubating cells with 0.1% saponin was the most effective for permeabilization of *T*. *brucei* cells. This is possibly due to large differences in parasite membrane composition, as in *T*. *brucei* the membrane contains a dense coat of Variant Surface Glycoprotein (VSG). While we have not rigorously tested whether increasing the exposure time to saponin influences permeabilization, we recommend that the saponin permeabilization step be completed in smaller batches of samples if large numbers of samples (>10) are being processed simultaneously.

The use of an anti-H3 antibody also allowed us to assess whether the protocol was working by looking for a characteristic 150bp ladder pattern that results when CUT&RUN is performed on histone proteins [9,16]. We tested different incubation times and temperatures for the MNase step of the protocol [16] and concluded that 5 minutes at room temperature allowed for efficient cutting while minimizing non-specific cutting events that can occur when incubation is performed at 37°C, as mentioned in Skene et al [9]. Thus, we recommend the use of an anti-H3 antibody as a first pass to assess success of the protocol. Although we designed the flow cytometry assay to optimize the permeabilization conditions for *T*. *brucei* cells, we also used it when performing CUT&RUN with Bdf3 as an early indicator for any problems with permeabilization while performing the assay, and we recommend that adopters of the protocol do this as well, particularly while troubleshooting. We did not test the use of concanavalin A beads during the procedure because we were concerned that VSG shedding might titrate the beads away from the parasite surface/nuclei. The use of concanavalin A beads could be tested in future iterations of the experiment.

Here we present a detailed protocol to perform CUT&RUN in *T*. *brucei* parasites. Our hope is that the use of this protocol may streamline the workflow for researchers interested in measuring genomic occupancy for proteins of interest. However, we caution users to be aware that large protein complexes are unable to diffuse out of the nucleus. In the protocol presented here, DNA is harvested from the supernatant following diffusion of the protein complex out of the nuclei. Thus, users working with large complexes may have to adapt the protocol presented here if they are working with large complexes, as previously described [9,22]. This protocol is also appropriate for researchers hoping to avoid a crosslinking step in their workflow and those who do not have access to a sonicator. Finally, other researchers hoping to adopt the protocol to a new model organism may find the flow cytometry assay helpful for optimizing the permeabilization step.

## Materials and methods

The protocol described in this peer-reviewed article is published on protocols.io (DOI: dx.doi.org/10.17504/protocols.io.x54v9p8ppg3e/v1 and is included for printing as S1 File with this article.

### Expected results

**Fig 1:**
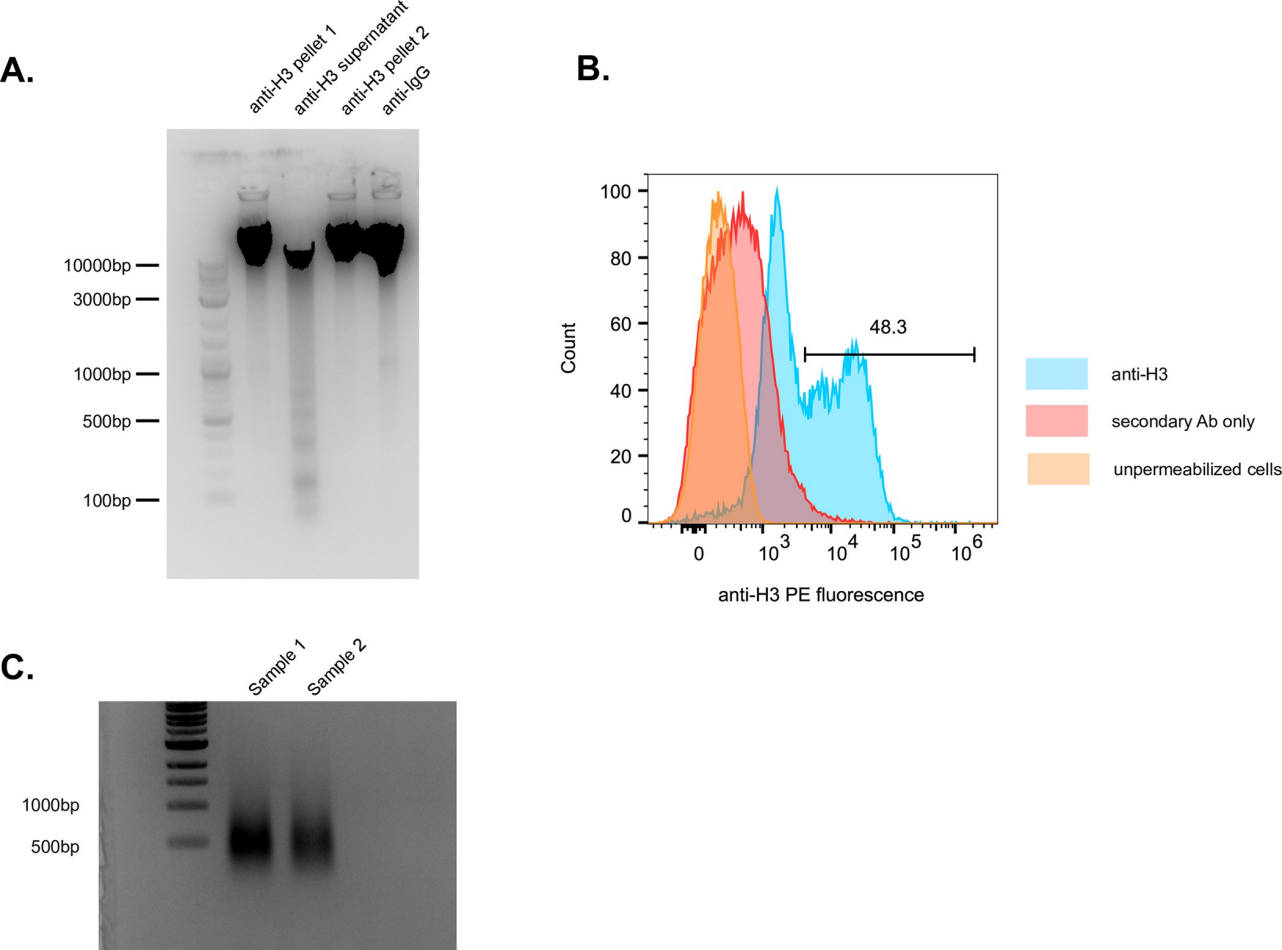
Expected CUT&RUN processing results. **(A)** Gel electrophoresis of DNA fragments processed using CUT& RUN. After incubation with anti-H3 and cleavage with pA-MNase the sample was separated into 2 pellet fractions and 1 supernatant fraction. All of the DNA harvested from 75 million cells was purified from each fraction and run in the lanes labeled anti-H3 pellet 1, anti-H3 supernatant, and anti-H3 pellet 2. The two pellet lanes were from the same sample split in half to facilitate loading. The characteristic ladder of ~150bp fragments is observed in the supernatant fraction. An additional sample was incubated with anti-IgG as a control (Lane anti-IgG). **(B)** Flow cytometry plot of permeabilized parasite cells incubated first with anti-H3 and then with anti-IgG PE (phycoerythrin, blue). Unpermeabilized parasites are shown in orange and parasites stained with anti-IgG PE alone (secondary Ab) are shown in red. The percent of positive staining cells is indicated. **(C)** DNA electrophoresis of two CUT&RUN sequencing libraries following PCR amplification. Sizes for the DNA ladder are indicated on the left. Uncropped images from this figure are available in S1 Fig.

We recommend optimizing CUT&RUN for an individual laboratory using an anti-H3 antibody, as this produces sufficient cut material to observe using an ethidium-stained DNA gel. Note that we used pA-MNase protein generously gifted to us by the Henikoff lab, but this has been superseded by pAG-MNase. The lane for supernatant harvested from nuclei following incubation with an anti-H3 antibody and cleavage with pA-MNase shows a typical pattern of fragments generated in ~150bp increments (anti-H3 supernatant lane, Fig 1A). This pattern of fragmentation is not observed when DNA is purified from the nuclear pellet (anti-H3 pellet 1 and anti-H3 pellet 2 lanes, Fig 1A) or when an anti-IgG antibody is used rather than an anti-H3 antibody (anti-IgG lane, Fig 1A). Flow cytometry analysis of the sample shown in Fig 1A reveals a strong positive fluorescent signal following incubation with an anti-IgG PE (phycoerythrin) labeled antibody (Fig 1B). Because the protein of interest may not be as ubiquitously distributed on the DNA, we recommend running an anti-H3 sample as a positive control when performing CUT&RUN, especially as the protocol gets established in an individual laboratory.

**Fig 2:**
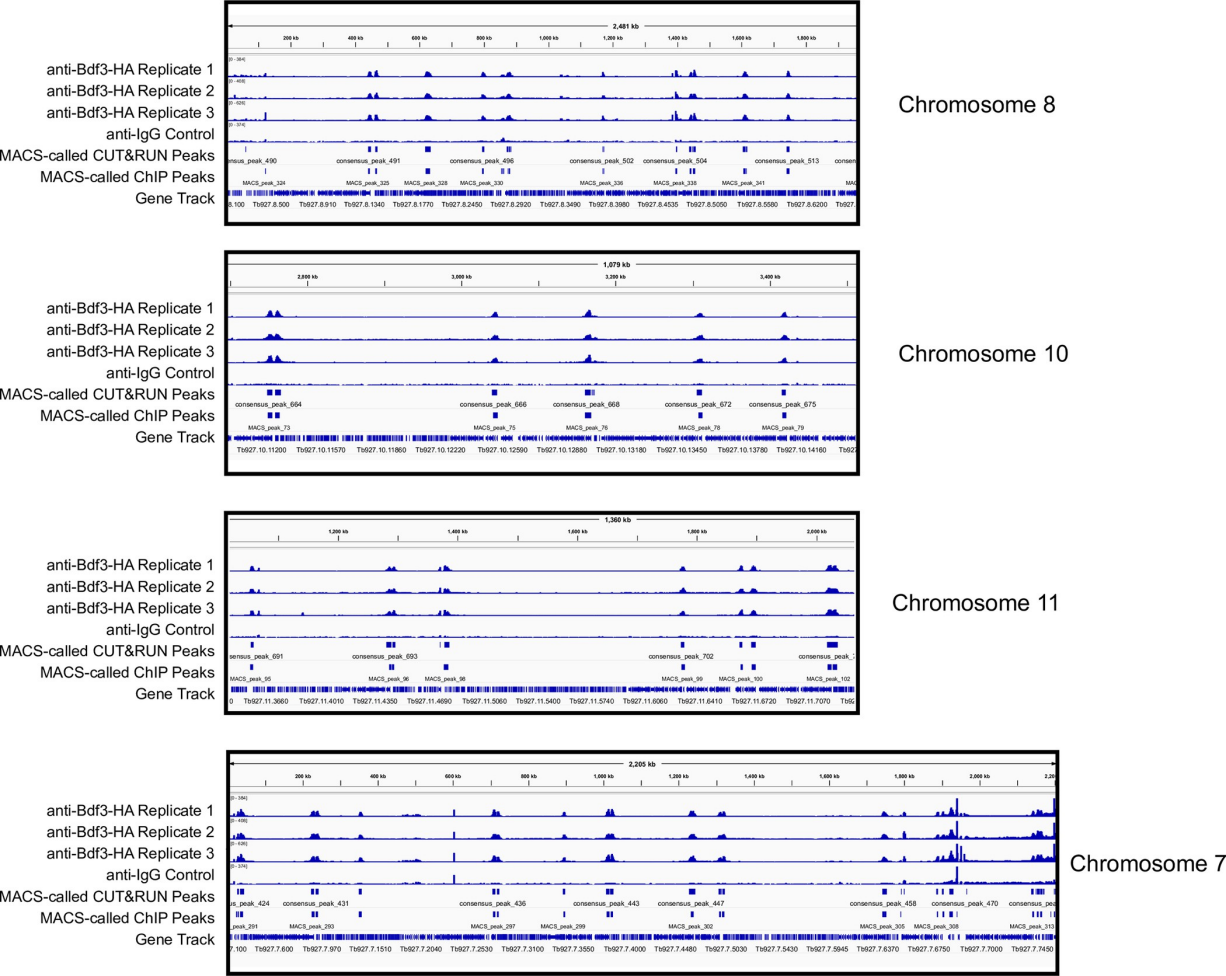
Results for BDF3 CUT&RUN. IGV traces for 4 different chromosomes showing three replicates processed by CUT&RUN using anti-HA in a bloodstream parasite line with HA-tagged Bdf3 [16]. An anti-IgG processed sample is shown as a control. CUT&RUN MACS [23] called peaks are also shown along with ChIP MACS-called peaks as a comparison.

Sequencing libraries for our samples were generated using the NEBNext Ultra II DNA Library Prep Kit for Illumina (E7645) according to the manufacturer’s instructions with the following modification: Samples were incubated with USER enzyme immediately prior to PCR, rather than at an earlier step. NEBNext Multiplex Oligos for Illumina were used to prepare multiplex samples (e.g. E7710). Typical DNA concentrations following PCR amplification of the libraries range from 70-250ng/μl. Representative examples of amplified DNA sequencing libraries are shown in Fig 1C. Data were analyzed as described in Ashby et al [16]. We sequenced our libraries on an Illumina HISEQ 3000 and obtained between 2.3*10^7^−3.7*10^7^ reads per library. An example of an IGV trace for anti-Bdf3-HA samples processed by CUT&RUN is shown in Fig 2. The trace shows obvious peaks of Bdf3 occupancy that are not observed in the control (Fig 2).

We calculated the Fraction of Reads In Peaks (FRIP) score for the three samples shown in Fig 2 and obtained scores of 0.31, 0.34, and 0.36. These scores are above the ENCODE standard of >0.1 for ChIP-seq [24]. Another lab that performed CUT&RUN on transcription factors in a *C*. *albicans* system obtained scores between 6 and 23% [25]. In a mammalian B cell system, Boyd et al obtained a FRIP score of 0.13 for the transcription factor Ikaros [26]. Thus, our signal to noise ratio is comparable with other CUT&RUN experiments in other systems.

### Limitations and potential shortcomings

As described above, the protocol presented here is for small protein complexes that can diffuse out of the parasite nucleus. The protocol would need to be modified to accommodate larger complexes that cannot diffuse out easily. In addition, proteins that bind DNA via transient interactions may be difficult to detect without crosslinking to trap the complex on the DNA. For proteins that transiently interact with DNA, ChIP-seq might be a more appropriate method because it includes a crosslinking step.

## Supporting information

S1 File Step-by-step protocol, also available on protocols.io. (PDF)

S1 Fig Uncropped gel images from Fig 1. (TIF)
