# Supplementary material for: Adaptation of CUT&RUN for use in African trypanosomes
Source: PLoS One. 2023 Nov 21;18(11):e0292784. doi: 10.1371/journal.pone.0292784 (PMC10662711; doi:10.1371/journal.pone.0292784)
Supplement: S1 File — (PDF) [file pone.0292784.s001.pdf]

CZDHX236

Cleavage Under Targets and Release Using Nuclease (CUT&RUN) V.(czdhx236)

Geneva Miller[1], Lindsey M. Rollosson[1], Carrie Saada[1], Serenity J. Wade[1], Danae Schulz[1]

[1]Harvey Mudd College, Claremont, CA

danaeschulz

Protocol Info: Geneva Miller, Lindsey M. Rollosson, Carrie Saada, Serenity J. Wade, Danae Schulz . Cleavage Under Targets and Release Using Nuclease (CUT&RUN). **protocols.io** https://protocols.io/view/cleavage-under-targets-and-release-using-nuclease-czdhx236Version created by danaeschulz

Created: Aug 30, 2023

Last Modified: Aug 30, 2023

PROTOCOL integer ID: 87177

Keywords: Trypanosoma brucei, CUT&RUN, genomic occupancy, mapping genomic protein binding sites

ABSTRACT

This Cleavage Under Targets and Release Using Nuclease (CUT&RUN) protocol produces genomic occupancy data for a protein of interest in the protozoan parasite *Trypanosoma brucei*. The data produced is analyzed in a similar way as that produced by ChIP-seq. While we describe the protocol for parasites carrying an epitope tag for the protein of interest, antibodies against the native protein could be used for the same purpose.

ATTACHMENTS

720-1571.docx

GUIDELINES

Guidelines

Pay extra attention to time-sensitive incubation periods, particularly the DNA cleavage step. We find that working through the protocol rather speedily produces the best results, so don't dawdle.

Additional Notes

We keep our centrifuges at 10 °C to make sure the samples don't freeze if the temperature drifts slightly. All spin steps of the protocol can be performed anywhere between 4 °C and 10 °C.

While we have not rigorously tested whether increasing the exposure time to saponin influences permeabilization, we recommend that the saponin permeabilization step be completed in smaller batches of samples if large numbers of samples
(>10) are being processed simultaneously.

MATERIALS

Materials

1. Spermidine trihydrochloride **Merck MilliporeSigma (Sigma-Aldrich) Catalog #S2501**
2. Saponin **Merck Millipore (EMD Millipore) Catalog #558255**
3. Sodium Chloride **Merck MilliporeSigma (Sigma-Aldrich) Catalog #S9888**
4. Calcium chloride **Merck MilliporeSigma (Sigma-Aldrich) Catalog #C4901**
5. Tris Hydrochloride (Tris-HCl) **Merck MilliporeSigma (Sigma-Aldrich) Catalog #RES3098T-B7**
6. cOmplete™ EDTA-free Protease Inhibitor Cocktail **Merck MilliporeSigma (Sigma-Aldrich) Catalog #11873580001**
7. UltrapPure 0.5M EDTA pH 8.0 **Invitrogen - Thermo Fisher Catalog #15575020**
8. Bioworld EGTA Buffer 0.5M pH 8.0 100ml **Fisher Scientific Catalog #50-255-957**
9. mouse anti-rabbit IgG-PE **Santa Cruz Biotechnology Catalog #sc-3753**
10. Anti-HA antibody produced in rabbit **Merck MilliporeSigma (Sigma-Aldrich) Catalog #H6908**

    , primary antibody will vary depending on your system
11. Rabbit anti-H3 (gift)
12. Sodium dodecyl sulfate solution **Merck MilliporeSigma (Sigma-Aldrich) Catalog #428018**
13. Proteinase K **Thermo Fisher Scientific Catalog #EO0491**
14. Monarch RNase A – 1 ml (2x0.5ml) **New England Biolabs Catalog #T3018L**
15. Ampure XP beads **Beckman Coulter Catalog #A63881**
16. CUT&RUN pAG-MNase and Spike-In DNA **Cell Signaling Technology Catalog #40366**

Equipment

1. Microcentrifuge (Eppendorf 5424R)
2. Swinging bucket centrifuge (Eppendorf 5920R)
3. 1.7ml Olympus microtubes (Genesee 22-281)
4. Olympus 50ml Centrifuge Tubes (Genesee 21-108)
5. Roto-Mini Plus Variable Speed Rotator with tube holders, 115V (ThermoFisher Scientific, 1159P34)
6. Novocyte Flow Cytometer 2000

Buffers

NP-S Buffer with 0.1% Saponin

A	B
Individual Components	
spermidine	0.5 mM
Saponin	0.1% (vol/vol)
NaCl	50 mM
Tris-Cl (pH 7.5)	10 mM
Store at 4ºC	
Add protease inhibitors just before use.	

NP-S Buffer no detergent

A	B
Individual Components	
spermidine	0.5 mM
NaCl	50 mM
Tris-Cl (pH 7.5)	10 mM
Store at 4ºC	
Add protease inhibitors just before use.	

Antibody Buffer

NP-S Buffer with 2mM EDTA

2X Stop Buffer

A	B
EDTA	20mM
EGTA	40mM
Add 50 pg of yeast spike-in DNA to each reaction, or 50 pg/100 µl of 2X Stop Buffer.	

BEFORE START INSTRUCTIONS

Before Start

We use 50-75 million bloodstream parasites per sample. Parasites are cultured in HMI9 media with incubation at 37 °C and 5% CO_2. Cultures should be prepared in advance so that sufficient numbers of parasites are available for each sample. The protocol works best when everything is kept cold prior to cutting with the protein A-MNase fusion protein. We recommend keeping buffers chilled on ice and pre-cooling centrifuges to 4-10 °C . Protease inhibitors should be added to the NP-S buffer just before commencing the experiment. The amount of **2X Stop Buffer** required for the experiment should be calculated and yeast spike-in DNA should be added prior to starting (see 2X Stop Buffer recipe below).

Prepare cells

1 Count parasite cultures with hemocytometer or other preferred counting method.

2 Spin down cells in centrifuge at 2800 x g, 00:10:00 . 10m

Note

This spin step can be performed at Room temperature or at 10 °C .

2.1 Remove supernatant and resuspend in small amount of remaining media (~ 100 µL).

3 If needed, combine samples from multiple Eppendorf tubes so that each final tube has 75 million cells, and spin again at 2800 x g, 10°C, 00:04:00 in microcentrifuge. Remove supernatant. 4m

Permeabilize cells

4 Wash all samples with 1 mL **NP-S Buffer with 0.1% Saponin.**

5 Spin at 4600 x g, 10°C, 00:04:00 . Remove sup. 4m

Primary Antibody Binding

6 Resuspend each sample in 100 µL **NP-S Buffer with 0.1% Saponin.**

7 Add EDTA to [M] 2 millimolar (mM) final (4 µL of [M] 0.05 Molarity (M) EDTA/ tube).

8 Add 5 µg α-HA antibody (or other antibody against protein of interest for experimental sample) OR add 5 µg α-H3 antibody (for control sample, might require titration) OR add 5 µg α-IgG antibody (for control sample).

9 Incubate for 00:45:00 on tube rotator at Room temperature. 45m

10 Add 1 mL NP-S Buffer no detergent to each sample.

11 Spin at 4600 x g, 10°C, 00:04:00. Remove supernatant. 4m

12 Add 1 mL NP-S Buffer no detergent to each sample.

13 Take out aliquot of 1 million cells for flow cytometry analysis (~ 14 µL if starting with 75 million cells). Set aside On ice.

14 Spin the remainder of the sample at 4600 x g, 10°C, 00:04:00. Remove supernatant. (total 2 washes). 4m

pAG-MNase binding

15 Resuspend each sample in 100 µL of buffer **NP-S Buffer no detergent.**

16 Add 1.5 µL of pAG-MNase enzyme to each sample.

17 Incubate for 00:45:00 at Room temperature on rotator. 45m

FACS Sample preparation

18 Add 1 mL **NP-S Buffer no detergent** to 1 million cell aliquot prepared above.

Note

We also routinely use HMI-9 media for the flow cytometry antibody incubation and washes.

19 Spin at 4600 x g, 00:04:00 . 4m

20 Resuspend in 100 µL NP-S Buffer no detergent.

21 Stain with α-rabbit IgG PE at 1:200 for 00:15:00 at Room temperature . 15m

22 Wash.

22.1 Wash in 1 mL NP-S Buffer no detergent (or HMI-9) at 7000 rpm, 00:04:00 . (1/2) 4m

22.2 Wash in 1 mL NP-S Buffer no detergent (or HMI-9) at 7000 rpm, 00:04:00 .(2/2) 4m

23 Resuspend in 300 µL NP-S Buffer no detergent (or HMI-9).

24 Transfer sample into flow cytometry tube.

25 Analyze on flow cytometer.

pAG-MNAse wash

26

Note

continued after 45m incubation above.

Add 1 mL **NP-S Buffer no detergent** to each sample.

27 Spin at 4600 x g, 10°C, 00:04:00 . Remove supernatant. 4m

28 Add 1 mL NP-S Buffer no detergent to each sample.

29 Spin at 4600 x g, 10°C, 00:04:00 . Remove supernatant. 4m

Targeted Digestion Preparation

30 Make sure to prepare enough **2X Stopbuffer** with spike in control.

Note

This is the section where timing is very important.

Targeted Digestion

31 Resuspend each sample in 100 µL buffer **NP-S Buffer no detergent.**

32 Incubate at Room temperature for 00:05:00 . 5m

33 Add 2 µL [M] 100 millimolar (mM) $CaCl_2$ to all samples (final concentration = 2mM), mix by flicking.

34 Incubate all samples at 25 °C for 00:05:00. 5m

Note

We recommend using a heat block as ambient temperature in the lab can vary.

35 Add 100 µL 2X Stop buffer to each sample and mix by flicking.

Chromatin Release

36 Incubate 00:10:00 at 37 °C to release CUT&RUN fragments from the insoluble nuclear chromatin. 10m

37 Spin at 4600 x g, 10°C, 00:04:00. Remove supernatant into new tube. SAVE SUPERNATANT. 4m

DNA Extraction

10m

38 To all samples add 2 µL of 10% SDS (final concentration = 0.1%), 3.3 µL of 10 undetermined proteinase K (165µg/ml), and 1.33 µL of 1 undetermined RNAse A (6.5µg/ml).

39 Mix by gentle flicking and incubate for 00:10:00 at 70 °C. 10m

40 Purify using Ampure XP beads at 1.8X or phenol chloroform extraction.

Note

Following DNA purification, high-throughput sequencing libraries can be prepared using the preferred method of the research lab.
