# Supplementary figures and images for: Adaptation of CUT&RUN for use in African trypanosomes
Source: PLoS One. 2023 Nov 21;18(11):e0292784. doi: 10.1371/journal.pone.0292784 (PMC10662711; doi:10.1371/journal.pone.0292784)

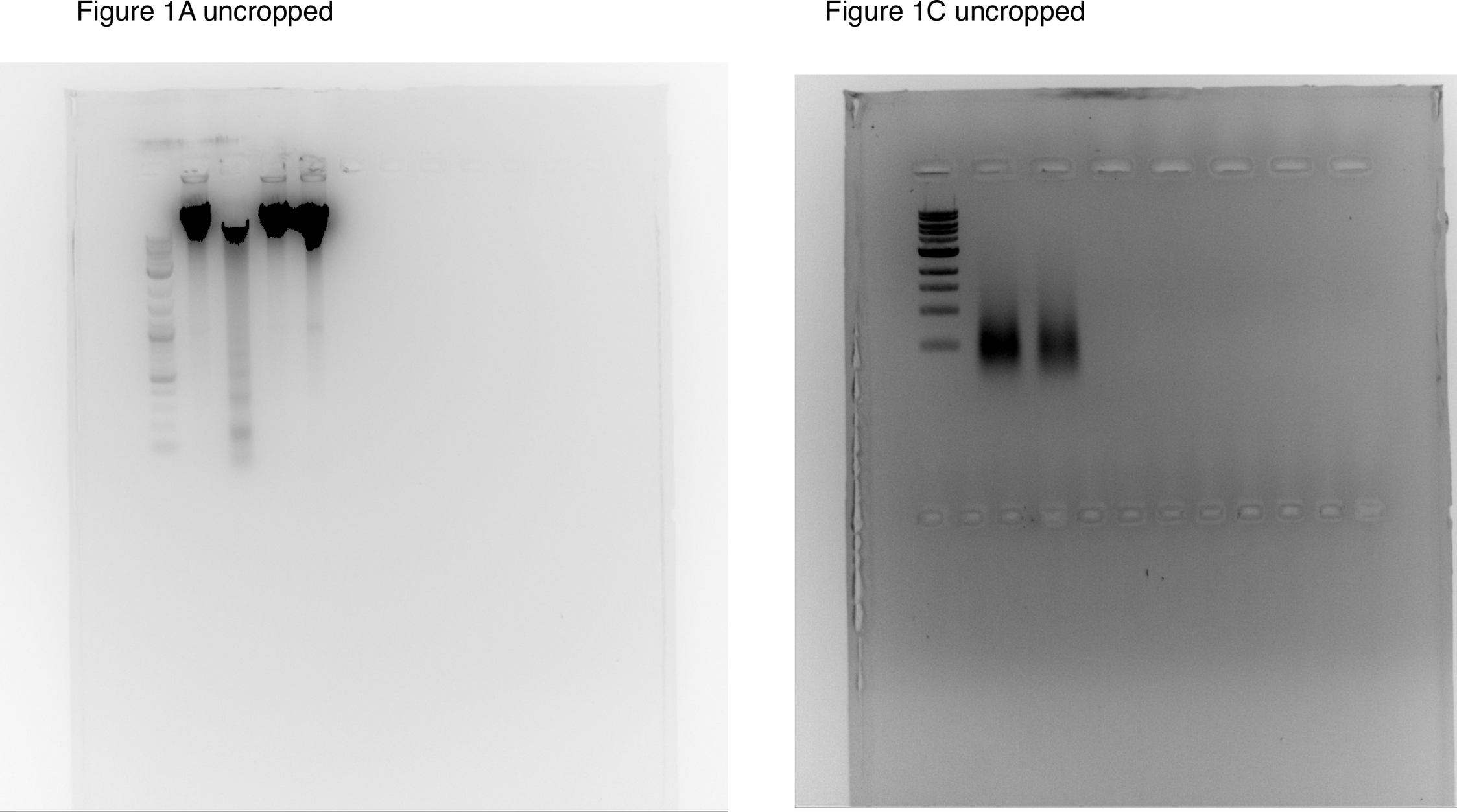

Supplement: S1 Fig — (TIF) [file pone.0292784.s002.tif]
